# Association between Love Breakup and Suicidal Ideation in Peruvian Medical Students: A Cross-Sectional Study during the COVID-19 Pandemic

**DOI:** 10.21203/rs.3.rs-3085268/v1

**Published:** 2023-07-19

**Authors:** Danai Valladares-Garrido, J. Pierre Zila-Velasque, Flor M. Santander-Hernández, Miguel A. Guevara-Morales, Noelia Morocho-Alburqueque, Virgilio E. Failoc-Rojas, César J. Pereira-Victorio, Víctor J. Vera-Ponce, Darwin A. León-Figueroa, Mario J. Valladares-Garrido

**Affiliations:** Universidad Cesar Vallejo; Universidad Nacional Daniel Alcides Carrion; Universidad Cesar Vallejo; Universidad Cesar Vallejo; Universidad Nacional de Piura; Universidad San Ignacio de Loyola; Universidad Continental; Universidad Ricardo Palma; Universidad de San Martín de Porres; South American Center for Education and Research in Public Health, Universidad Norbert Wiener

**Keywords:** medical students, mental health, suicidal ideation, COVID-19, pandemic, Peru

## Abstract

**Objective:**

We aimed to determine the association between a major romantic breakup and suicidal ideation in medical students from three universities in Peru.

**Methods:**

A cross-sectional study was conducted during the first pandemic wave in 2021 on medical students from three universities in northern Peru. The outcome was suicidal ideation, measured with question nine of the PHQ-9. The exposure was the experience of a major love breakup during the pandemic. In addition, its association with other covariates (age, sex, family members infected with COVID-19, deceased family members with COVID-19, insomnia, and anxiety, among others) was examined.

**Results and discussions:**

Out of 370 students, 19.5% reported a major love breakup during the pandemic (95%CI: 15.5–23.8), and 34.3% had suicidal ideation (95%CI: 29.4–39.4). Having a major love breakup was associated with a higher prevalence of suicidal ideation (PR: 1.49, 95%CI: 1.32–1.67). Moderate insomnia (PR: 2.56, 95%CI: 1.70–3.87) and anxiety symptoms (PR: 1.94, 1.10–3.44) were also associated with suicidal ideation.

**Conclusion:**

Our study provides evidence of a significant association between a major love breakup and suicidal ideation. This finding emphasizes the need for further research to better understand this association and inform the development of effective suicide prevention policies in medical education.

## 1 Introduction

Worldwide, suicide is responsible for more than 700,000 deaths annually and is the fourth leading cause of death in young people aged 15–29 years, with a higher percentage of deaths in low- and middle-income countries (1). Suicidal ideation (SI) is considered the most important risk factor for suicide (1, 2), a public health problem that has recently begun to be studied in Latin America (3).

Medical students have a prevalence of SI from 13.7–33.3% (4–6). Due to the COVID-19 pandemic, not only was physical health affected (7–9), but mental health has also deteriorated significantly in various populations (10–15). Similarly, in the context of the COVID-19 pandemic, suicidal ideation increased from 13.4–16.9%, according to a study of Mexican students (16), a result similar to a French study (17). In Peru, 17.9% of medical students had SI during the COVID-19 pandemic, which was found to be associated with a higher frequency of depression and anxiety (18). Additionally, the development of this disease is influenced by several potential protective factors: the search for psychological help or support in their family (19), no mental health disorders and/or use of illegal substances (20), and to have stability in their love relationship (21, 22). Conversely, having depression, poor social support, less frequent conversations, and ending a stable romantic relationship all behave as aggravating risk factors for the development of SI (5, 23–25).

Here we highlight a variable that has been focused on in groups other than medical students (5, 20–22, 26), as is the termination or breakup of a love relationship. Relationships are one of the most important social interactions for human beings, and their termination can cause physical and mental problems, such as suicidal thoughts and behaviors (27, 28). The impact of rupture is even greater in young people such as medical students (29). The breakdown of romantic relationships has increased during the pandemic due to government-imposed restrictions such as social distancing that led to staying at home [30] and actions not unrelated to Peru (30, 31). Results have been found in a higher proportion of women and health professionals (32) and even more so in the university population that has undergone curricular changes that have led to the development of mental health and stress disorders (33).

However, there is no conclusive evidence in medical students that the psychological impact of a breakup is compounded by the physical and mental exhaustion produced by the academic load (26, 33), which would lead to an increased presence of SI (1). In addition, the studies that evaluated these variables had confounding and information biases because they did not include variables that have been shown to be associated with the outcome, such as mental health disorders, and because they were measured with instruments that have not been validated in the context of the study population (22, 34, 35). Finally, the studies were conducted on smaller samples (16, 22, 36). Therefore, in the present study, we evaluated whether love breakup influences the presence of SI, together with other variables of interest such as insomnia, a history of having a family member who died from COVID-19 and obesity, variables not included in previous studies, and that could potentially act as confounders (5, 18).

## 2 Methods

### Study Design

We conducted an analytical cross-sectional study based on a secondary analysis of a study assessing the association between excessive phone use and mental health disorders (37). This study was carried out among medical students in Piura between July and October 2020, a time when Peru was experiencing the first COVID-19 wave and with the restrictive measures imposed by the government to limit the increase in the number of infections. The present study aimed to evaluate whether a love breakup is associated with suicidal ideation in medical students.

### Population and Sample

The population consisted of medical students over 18 years of age who completed and accepted the informed consent form and responded to the survey. The inclusion criteria for the primary study included students who had a cell phone with permanent access to the internet for their activities. The exclusion criteria for the primary study included self-reporting of a diagnosis of a mental health disorder (anxiety and depression). For the primary study, a sample of 370 students corresponding to three universities was captured. The study selected participants by convenience sampling.

### Procedure

Prior to data collection, authorization was requested from the three participating universities. Subsequently, the research was conducted from July to October 2020, in the context of the first COVID-19 pandemic wave, when university higher education was providing virtual teaching in Peru. The form was designed and reviewed in Google Forms, then disseminated to the official social network groups of each year of study of the participating medical schools. The questionnaire was shared at times when the students were not in evaluations, and the approximate duration was 10 min. The questionnaire consisted of (1) informed consent, (2) socio-educational data, and (3) mental health data (PHQ-9, GAD-7), including insomnia (ISI). Finally, the data were exported from Google Forms to a database in Microsoft Excel, which underwent strict quality control prior to statistical analysis.

### Instruments

#### Patient Health Questionnaire-9 (PHQ-9)

An instrument that was validated in Peru, consisting of nine items with questions related to the presence of depressive symptoms in the last two weeks, evaluated on a Likert-type scale (38). The instrument has presented sensitivity and specificity values of 88% and 92%, respectively, in addition to a Cronbach’s alpha consistency of 0.84 (39). In this research, the last item of the PHQ-9 was used to measure suicidal ideation (“how often have you been bothered during the past 2 weeks by thoughts that you would be better off dead or thoughts of hurting yourself in some way?”) (38).

#### Generalized Anxiety Disorder Scale-7 (GAD-7)

An instrument that evaluates the presence of anxiety symptoms. It consists of seven items evaluated on a Likert-type scale (40). The instrument has shown sensitivity and specificity values of 89% and 82%, respectively (40), in addition to a Cronbach’s alpha consistency of 0.89 (41).

#### Insomnia Severity Index (ISI)

An instrument that assesses the presence of insomnia symptoms by means of an instrument that evaluates seven items, an instrument that has been validated in Spanish (42), with a sensitivity and specificity of 86.1% and 87.7%, respectively (43), in addition to a Cronbach’s alpha consistency of 0.84(44).

#### Sociodemographic and educational Data

age in years, sex, single marital status (no, yes), obesity (no, yes; based on body mass index calculation using self-reported weight and height), self-report of having had a family member infected (no, yes) and deceased by COVID-19 (no, yes), and self-report of having suffered a serious economic problem in the last 3 months (no, yes).

### Variables

The dependent variable was suicidal ideation, defined as a student’s response to question 9 of PHQ-9. The question assesses whether respondents had thoughts that they would prefer to be dead or to harm themselves in some way. The initial responses were no days, several days, more than half of the days, and almost every day. For the analysis of this research, it was dichotomized into no and yes (several days-almost every day).

The primary independent variable was major love breakup, defined as a student’s self-report of having suffered a major relationship breakup during the COVID-19 pandemic (no, yes).

Secondary independent variables were age in years, sex (female, male), single (no, yes), having obesity (no, yes), report of having had a close relative with COVID-19 (no, yes), report of having had a deceased relative with COVID-19 (no, yes), report of having suffered a serious financial problem in the past 3 months (no, yes), insomnia (no, below the threshold, moderate, severe), and anxious symptoms (no, yes).

### Statistical Analysis

#### The statistical analysis was performed in Stata 16.1.

For descriptive analysis, we showed absolute and relative frequencies for categorical variables. For numerical variables, we evaluated the assumption of normal distribution and then reported the best measure of central tendency and dispersion.

For the bivariate analysis, the association of interest (love breakup vs. suicidal ideation) was evaluated, as well as the rest of the categorical covariates, through the chi-square test of independence. In the case of numerical variables, the Mann–Whitney U test was useful after evaluating the assumption of normal distribution. The significance level was 5%.

For the simple and multiple regression analysis, we used generalized linear models with Poisson distribution, robust variance, and universities as groups or clusters. This allowed us to estimate prevalence ratios (PR) and 95% confidence intervals (95%CI) for the association of interest and the rest of the exposure variables. In the multiple regression analysis, confounding variables served as a model adjustment to assess the association between love breakup and suicidal ideation. Collinearity between the variables of interest was assessed with the variance inflation factor, giving an overall estimate of 2.17.

### Ethical Aspects

The primary study was approved by the Ethics Committee of the Universidad Cesar Vallejo, Piura, Peru. The questionnaires were anonymous, and informed consent was obtained from all participants prior to their participation in the research, in accordance with the ethical procedures established by the Ethics Committee of Cesar Vallejo University (Piura, Peru). The ethical principles of the Declaration of Helsinki were maintained.

## 3 Results

### Characteristics of the Participants

Of the 370 students analyzed, 61.9% were male, and the median age was 20 years (19–23); 7.6% were obese, 20.5% had moderate insomnia, and 68.9% had anxious symptoms; 19.5% reported having had a major love breakup during the pandemic (95%CI: 15.55–23.87); 34.3% of the students presented suicidal ideation (95%CI: 29.49–39.41) (Table 1).

### Love Breakup and Other Factors Associated with Suicidal Ideation, in Bivariate Analysis

Students who reported having a major love breakup during the pandemic had a 24.7% higher frequency of suicidal ideation compared to students who did not have a love problem (54.2% vs. 29.5%; *p* < 0.001). Having anxious symptoms increased the frequency of suicidal ideation in students by 35.9% compared to those without anxiety (45.5% vs. 9.6; *p* < 0.001). Students with severe insomnia had a 53.2% higher frequency of suicidal ideation compared to those without sleep problems (66.7% vs. 13.5%; *p* = 0.001). Additionally, age (*p* = 0.003), obesity (*p* < 0.001), having a family member with COVID-19 (*p* = 0.008), having had a deceased family member with COVID-19 (*p* = 0.020) were significantly associated with having suicidal ideation in the evaluated students (Table 2).

### Love Breakup and Other Factors Associated with Suicidal Ideation in Simple and Multiple Regression Analyses

Table 3 shows the simple and multiple regression analyses. The simple regression model showed that students with strong love breakups during the pandemic had an 83% higher prevalence of suicidal ideation (PR: 1.83; 95%CI: 1.60–2.10). This was maintained in the multiple regression model in terms of direction and magnitude: having a major romantic breakup increased the prevalence of suicidal ideation by 49% (PR: 1.49; 95%CI: 1.32–1.67).

Having moderate insomnia increased 156% the prevalence of suicidal ideation (PR: 2.56; 95%CI: 1.70–3.87). Students with anxious symptoms had a 94% higher prevalence of suicidal ideation (PR: 1.10–3.44). Additionally, for each additional year of age, students had a 3% higher prevalence of suicidal ideation (PR: 1.03; 95%CI: 1.01–1.05). Female students had an 11% lower prevalence of suicidal ideation (PR: 0.89; 95%CI: 0.80–0.99) (Table 3).

## 4 Discussion

### Prevalence of Suicidal Ideation in Medical Students

In this study, we found that the prevalence of SI was 34.3% (three out of 10 students). Our result differs from that reported in studies conducted in the pre-pandemic context, such as in Ecuador, conducted in medical and psychology students, which identified a prevalence of severe SI of 4.5%, moderate in 19.1% and mild in 76.4%. It should be noted that the different results could be due to the use of a different instrument, such as the ISO-30 (Inventory of Suicide Orientations) (45). Similarly, in Colombian medical students, it was found that the SI was 17.7% (46) and in Mexico, 8.7% after surveying undergraduate and graduate students (3). Likewise, in Peru, a prevalence of SI of 11.2% has been identified in medical students to whom the MINI (Mini-International Neuropsychiatric Interview) test was applied (47). Similarly, other Peruvian studies on university students of other careers identified that SI was present in 8.9–35.2%. It should be noted that self-developed instruments were used for the detection of SI (48, 49). These results are supported by a meta-analysis that has shown that in Latin America, the prevalence of SI is 13.8%, lower than in Europe and the USA (3). Similarly, our result differs from those reported by medical students in other countries, such as Italy (4), Iran (50), and Ethiopia (5) (13.7, 17.0, and 23.7%, respectively). It should be noted that these studies were conducted in the pre-pandemic period.

Among the studies conducted in the context of the COVID-19 pandemic, our result also differs from that reported in undergraduate students at a U.S. public university in the second wave, where 12.7% of SI was reported (51). It also differs from what was found in medical students in Mexico, where an SI prevalence of 18.6% was found in the first wave of the pandemic (16). Likewise, it is higher than that found in medical students in Peru during the first pandemic wave, where a prevalence of 17.9% of SI was estimated (18). The disparate results could be due to the different moments of the application of the instrument since our study was at the beginning of the pandemic during the first wave, when the uncertainty and consequences in the near future caused fear and anxiety in the population, while the other study was conducted at the end of the second wave (51). At this point, we also highlight that no other Latin American study has been found in the pandemic context on the evaluation of SI, so our study adds data to the current literature.

In Peru, according to the report of the National Institute of Mental Health “Honorio Delgado-Hideyo Noguchi”, listed as the center of excellence in mental health and Psychiatry, a total of 3388 consultations for SI in people over 18 years old were recorded, with an increase of 43.2% in the monthly incidence of SI during the pandemic compared to the previous two years (2018–2019) (52). This supports the results found in our study, which could be explained by the fact that medical students were exposed to more stressors. In addition, it was conducted in a different geographical area and with a different instrument than those used in the aforementioned studies, such as the Beck Depression Instrument (BDI-II) (4), and the Depression, Anxiety and Stress Scale (DASS) (5).

### Frequency of Severe Love Breakup in Medical Students

The present study found that 19.5% (two out of 10) of the students reported having had a major romantic breakup in the last three months, lower than that reported by studies conducted in the pre-pandemic context by Espinosa et al. with a frequency of 46.9% of breakups in university students of various degrees of the FESI, UNAM in Mexico, possibly due to cultural differences, stress, anxiety, and lack of management of interpersonal problems. Similarly, it has been reported that 59.6% of university students in careers other than human medicine reported a romantic breakup (53). This difference could be explained by the fact that 66.5% of these students belonged to the psychology career, while the medical students came expressing stress, anxiety, and SI without mentioning any love breakup. Our result is different because it was carried out in the context of the pandemic; however, we did not find other studies that evaluate this variable in the context of the pandemic. The fact that at least two out of 10 students presented a strong romantic breakup during the pandemic could be explained because social distancing measures such as staying at home were established, a situation that led to the development of stress that was greater because confinement predisposes the couple to stay alone and that their problems are highlighted and cannot be solved due to the lack of social and work interactions (54).

### Association between Having Had a Major Breakup and Suicidal Ideation

Students who reported having had a major love breakup had a 38% higher prevalence of SI during the pandemic. This is similar to the findings of Tan et al. (23) in Malaysian medical students, one of the significant predictors of SI was breaking up a stable romantic relationship (OR: 5.4, 95%CI: 1.3–22.4). Furthermore, it is consistent with another study in which the prevalence of suicide was higher (17.0%) in Iranian medical students who were separated or divorced (50). On the other hand, Barajas Marquez (55) reported that university students who had had a recent love breakup presented a higher level of depression. This is consistent with what was reported prior to COVID-19 by Espinoza-Sierra et al., who stated that the main cause of university students visiting the crisis, emergency, and suicide care center (CREAS) is the breakup of a couple, followed by bereavement and relationship problems. While for McLaughlin and Gunnell (56), who collected information on deaths of university students in the United Kingdom between 2010 and 2018, it is worth noting that the study was conducted in a pre-pandemic context, the most influential factors in suicidal behavior were love breakups, failing subjects, economics and recent bereavement. This is also evidenced in a systematic review that reported that separation or love breakup represents the most important risk factor for the development of SI in young people aged 15–29 years (27). The association found differs from that reported by Kazan (27) in Australia during the pre-pandemic context, where it was reported that women especially experienced relief and benefited from ending an abusive or negative relationship. The latter study, however, was conducted in the general adult population and not in medical students like the present study (27) and explored the quality of the relationship rather than the specific event of a breakup. It is worth noting that we did not find other studies evaluating this association, particularly during the context of the COVID-19 pandemic. This association could be due to partner problems that behave as drivers of SI due to a lack of resilience (which has been frequently evidenced in the context of the pandemic), emotional control, and psychological support by institutions and the corresponding family support (57, 58).

### Other Factors Associated with Suicidal Ideation

Having insomnia was associated with an 85% higher prevalence of SI. This result is similar to that described before the pandemic by Liu et al. (59) in Chinese university students who presented 5 times the risk of suffering SI (OR = 4.98). This is consistent with that described by Khader et al. (60), who reported that university students with insomnia presented four times more SI in the pre-pandemic context. This is consistent with that described by King et al. (61), who conducted a study at a Canadian university and found a positive association between insomnia and suicidal ideation. Moreover, it is consistent with the findings of Akram et al. (62), who conducted a study in the pre-pandemic context and found a positive correlation between insomnia and suicidal ideation in American college students. This association is explained by different postulated mechanisms, such as the decrease or alteration of serotonin 5-HT receptors (an amino acid involved in the maintenance of sleep) in subjects with suicidal tendencies (63), nightmares (64) and the alteration of the hypothalamic-pituitary axis (65). However, its association remains under constant investigation, although bidirectionality has also been found because people with SI also have the subsequent development of insomnia between this association and insomnia (66).

For each additional year of age, the prevalence of SI increased by 3%. This is similar to that reported by Schwenk (67), who reported in his pre-pandemic study that third and fourth-year U.S. medical students reported a higher frequency (7.9%) of SI compared to first and second-year students (1.4%). However, for Khader (60), in his study carried out in the pre-pandemic context in Pakistan in university students of different careers, no positive significance was found between the university year completed and SI. This association could be due to the academic load that deals with the poor organization of time and clinical practice due to the perception of death and suffering of the hospitalized.

Female students had an 11% lower prevalence of SI. Our result differs from that reported by Osama et al. (68) and Schwenk et al. (67), who identified that those who present the highest prevalence of SI are women because they develop higher levels of moderate to severe depression (18.0%), a situation that leads them to think that having a mental health disorder could generate insecurity to give an opinion or suggestion and less capacity to fulfill their responsibilities. This association could be due to the fact that women have a greater social burden due to the expectation that they play the role of the person who provides stability in the home and the social role of a country with high percentages of machismo (69). According to the United Nations Organization, it translates into intimate partner violence in women between 15 and 49 years of age, with percentages ranging from 33.0–51% worldwide (70), in addition to the subjective emotional problems of image (71).

Having anxiety increased the probability of SI by nine times. This is similar to that reported by Xu et al. (72), who found that Chinese medical students in the first pandemic wave presented who had anxiety symptoms were more likely to have SI (OR = 1.66). In Peru, Crisol-Deza et al. (18) reported that SI was associated with a higher probability of anxiety in medical students during the first wave of the pandemic (OR: 2.01). This is consistent with that described by Asfaw et al. (5) in Ethiopian students in the pre-pandemic context, where anxiety and depression were associated with an increased likelihood of SI. We have not found a study that identifies depression as an attenuating factor for the development of SI. This association could be due to the characteristics of a university student, loneliness, social shelter, and economic limitations that increased during the context of the pandemic, as mentioned above, due to government restrictions to limit the increase in infections.

### Limitations and Strengths

Our study has important limitations. First, the cross-sectional study design does not allow us to establish causality between variables. Second, selection bias, since a convenience sample was taken, it is not possible to infer the results for the entire population of interest. Third, being a secondary data analysis study, there is an unmeasured confounding effect since potential confounders such as the level of resilience of the students and the level of family communication, which behave as predisposing factors to various mental health disorders, have not been investigated (73, 74).

However, the study presents strengths. First, to our knowledge, it is the first study to evaluate this association of variables conducted during the COVID-19 pandemic, particularly during the first wave, the most critical time in Peru (75–79). Second, it was possible to capture a broad and varied sample (years of study) of students, and it is reinforced by the stratified choice of the sample by the university, which will serve as an aid to the strategies of the corresponding institutions. Third, several validated and widely used instruments were used in the scientific field, in addition to using a timely methodology considering the mediation of intervening variables.

### Relevance of Findings in Mental Health

This study adds to the current literature an underexplored finding, which proposes a precedent for future research in mental health. The students surveyed show a significant prevalence of romantic breakups that might trigger suicidal ideation and suicide in the worst-case scenario. Our results could be the basis for driving possible implications at the public health level by promoting services that eliminate the detrimental aspects of love breakup through counseling and psychology sessions for the couple in university welfare centers. We believe that social and educational programs should be promoted that encourage critical reflection on SI with the aim of reversing its factors, among them the growing love breakup. We recommend that through the tutoring areas of each university, periodic evaluations should be carried out for early detection and management of suicidal behavior to develop coping strategies and solve these problems.

## 5 Conclusions

Three and two out of 10 medical students experienced SI and a major love breakup, respectively. Our main result suggests that experiencing a major love breakup might predispose to the development of SI. As secondary results, insomnia, anxiety, and being older in the university stage were associated with SI. We recommend the development of further research that clarifies the association between major love breakups and SI and that medical schools provide periodic evaluations of mental health for the timely prevention of suicide.

## Availability of data and Materials

The dataset generated and analyzed during the current study is not publicly available because the ethics committee has not provided permission/authorization to publicly share the data, but it is available from the corresponding author upon reasonable request.

## Figures and Tables

**Table 1:** Characteristics of participants (n = 370).

Characteristics	n (%)
**Age (years)** *	20 (19–23)
**Sex**	
Male	141 (38.1)
Female	229 (61.9)
**Academic year**	
First	68 (18.4)
Second	72 (19.6)
Third	70 (18.9)
Fourth	68 (18.4)
Fifth	40 (10.8)
Sixth	35 (9.5)
Seventh	17 (4.6)
**Single marital status**	
No	363 (98.1)
Yes	7 (1.9)
**Obesity**	
No	342 (92.4)
Yes	28 (7.6)
**Family member diagnosed with COVID-19**	
No	146 (39.5)
Yes	224 (60.5)
**Family member deceased due to COVID-19**	
No	276 (74.6)
Yes	94 (25.4)
**Financial hardship**	
No	247 (66.8)
**Characteristics**	n (%)
Yes	123 (33.2)
**Insomnia severity index**	
No	119 (32.2)
Subthreshold	169 (45.7)
Moderate	76 (20.5)
Severe	6 (1.6)
**Anxiety**	
No	115 (31.1)
Yes	255 (68.9)
**Love breakup during pandemic due to COVID-19**	
No	298 (80.5)
Yes	72 (19.5)
**Suicidal ideation**	
No	243 (65.7)
Yes	127 (34.3)

* Age expressed as median and p25–p75.

**Table 2:** Love breakup and other factors associated with suicidal ideation, bivariate analysis.

Variables	Suicidal Ideation		*p* *
	No (n = 243)	Yes (n = 127)	
	n (%)	n (%)	
Age (years) †**	20 (18–22)	21 (19–24)	**0.003**
Sex			0.137
Male	86 (61.0)	55 (39.0)	
Female	157 (68.6)	72 (31.4)	
Single marital status			0.631
No	4 (57.1)	3 (42.9)	
Yes	239 (65.8)	124 (34.2)	
Obesity			**<0.001**
No	234 (68.4)	108 (31.6)	
Yes	9 (32.1)	19 (67.9)	
Family member diseased by COVID-19			**0.008**
No	84 (57.5)	62 (42.5)	
Yes	159 (71.0)	65 (29.0)	
Family member died by COVID-19			**0.020**
No	172 (62.3)	104 (37.7)	
Yes	71 (75.5)	23 (24.5)	
Financial hardship			0.379
No	166 (67.2)	81 (32.8)	
Yes	77 (62.6)	46 (37.4)	
Insomnia			**0.001**
No	103 (86.6)	16 (13.4)	
Subthreshold	115 (68.1)	54 (31.9)	
Moderate	23 (30.3)	53 (69.7)	
Severe	2 (33.3)	4 (66.7)	
Anxiety			**<0.001**
No	104 (90.4)	11 (9.6)	
Yes	139 (54.5)	116 (45.5)	
Love breakup during pandemic due to COVID-19			**<0.001**
No	210 (70.5)	88 (29.5)	
Yes	33 (45.8)	39 (54.2)	

* p-value of categorical variables calculated with Chi-Square test.

** p-value of categorical-numerical variables calculated with the U-test (Mann-Whitney).

† Median—interquartile range. Bold numbers emphasize significant p-values (p < 0.005).

**Table 3:** Love breakup and other factors associated with suicidal ideation, regression analysis.

Characteristics	Suicidal Ideation									
	Simple Regression						Multiple Regression			
	PR		95%CI		*p* *		PR	95%CI		*p* *
Age (years)	1.05		1.01–1.10		**0.048**		1.03	1.01–1.05		**0.009**
Sex										
Male	Ref.						Ref.			
Female	0.81		0.55–1.18		0.272		0.89	0.80–0.99		**0.040**
Single marital status										
No	Ref.						Ref.			
Yes	0.80		0.26–2.45		0.692		1.12	0.21–6.01		0.892
Obesity										
No	Ref.						Ref.			
Yes	2.15		1.68–2.74		**<0.001**		1.25	0.79–1.97		0.350
Family member diseased by COVID-19										
No	Ref.						Ref.			
Yes	0.68		0.53–0.88		**0.003**		0.95	0.74–1.20		0.652
Family member died by COVID-19										
No	Ref.						Ref.			
Yes	0.65		0.37–1.15		0.138		0.70	0.44–1.10		0.121
Financial hardship										
No		Ref.				Ref,				
Yes		1.14	0.82–1.58	0.434		1.13			0.87–1.47	0.357
Insomnia										
No		Ref.				Ref.				
Subthreshold		2.38	1.40–4.03	0.001		1.58			0.94–2.64	0.084
Moderate		5.19	2.98–9.04	<0.001		2.56			1.70–3.87	**<0.001**
Severe		4.96	2.79–8.82	<0.001		1.93			0.86–4.37	0.112
Anxiety										
No		Ref.				Ref.				
Yes		4.76	2.76–8.20	<0.001		1.94			1.10–3.44	**0.023**
Love breakup during pandemic due to COVID-19										
No		Ref.				Ref.				
Yes		1.83	1.60–2.10	**<0.001**		1.49			1.32–1.67	**<0.001**

* Adjusted for age, sex, single marital status, obesity, family member diseased or dead by COVID-19, financial hardship, insomnia, and anxiety. Universities were set as clusters. Bold numbers emphasize significant p-values (p < 0.005).
